# Supplementary material for: Complexome profiling of the Chlamydomonas psb28 mutant reveals TEF5 as an early PSII assembly factor
Source: Plant Cell. 2025 Mar 17;37(6):koaf055. doi: 10.1093/plcell/koaf055 (PMC12164590; doi:10.1093/plcell/koaf055)
Supplement: koaf055_Supplementary_Data [file koaf055_supplementary_data.zip › psb28_interactive.zip!/plots/intensity/qualityPlots/peptides/NUO10_Cre12.g492300.t1.2.html]

### Cre12.g492300.t1.2

Trivial name: NUO10  
SeqCoverage [median]: 43.90  
Mass: 18.12 kDa  
  
 

| Sequence | MissedCleavage | Mass [Da] | wtIntensity | muIntensity | Score |
| --- | --- | --- | --- | --- | --- |
| GLEFVVSK | 0 | 877.49 | 22308.10 | 37697.52 | 116.25 |
| KVYDQMPEPK | 1 | 1233.61 | 4841.67 | 5039.23 | 98.04 |
| QSDAMIVAGTLTNK | 0 | 1447.73 | 16069.90 | 7028.41 | 234.71 |
| SKPAAAAK | 1 | 742.43 | 27973.75 | 35262.61 | 133.60 |
| TTQIWYK | 0 | 938.49 | 1148.93 | 1212.77 | 126.24 |
| TTQIWYKK | 1 | 1066.58 | 977.03 | 2779.89 | 57.18 |
| VDNVVNWAR | 0 | 1071.55 | 8890.73 | 3976.05 | 145.16 |
| VYDQMPEPK | 0 | 1105.51 | 4227.99 | 4131.74 | 149.65 |
| YDLDRYGIIFRPSPR | 2 | 1866.97 | 752.53 | 6878.89 | 37.55 |
| YGIIFRPSPR | 1 | 1204.67 | 16756.48 | 30081.49 | 85.38 |
|  |  |  | 103947.1 | 134088.6 |  |
